# Investigation of foot and mouth disease virus and other animal pathogens in cattle, buffaloes and goats at the interface with Akagera National Park 2017 – 2020

**DOI:** 10.1186/s12917-022-03430-1

**Published:** 2022-09-16

**Authors:** Jean Claude Udahemuka, Gabriel Aboge, George Obiero, Angélique Ingabire, Natasha Beeton, Evodie Uwibambe, Phiyani Lebea

**Affiliations:** 1 Department of Biochemistry, Centre for Biotechnology and Bioinformatics, University of Nairobi, P.O. Box 30197, Nairobi, Kenya; 2 Department of Veterinary Medicine, University of Rwanda, P.O. Box 57, Nyagatare, Rwanda; 3 Department of Public Health, Pharmacology and Toxicology, Faculty of Veterinary Medicine, University of Nairobi, P.O. Box 29053, Nairobi, Kenya; 4 Rwanda Agriculture and Animal Resources Board, P.O. Box 5016, Huye, Rwanda; 5 TokaBio (Pty), Ltd, Pretoria, South Africa

**Keywords:** FMD, RT-LAMP, RT-PCR, Buffaloes, FMDV, SAT 2, Seroprevalence, Rwanda

## Abstract

**Background:**

Foot-and-Mouth Disease Virus (FMDV) is a positive-sense RNA virus of the family of the picornaviridæ that is responsible for one of the livestock diseases with the highest economic impact, the Foot-and-Mouth Disease (FMD). FMD is endemic in Rwanda but there are gaps in knowing its seroprevalence and molecular epidemiology. This study reports the FMD seroprevalence and molecular characterization of FMDV in Eastern Rwanda.

**Results:**

The overall seroprevalence of FMD in the study area is at 9.36% in cattle and 2.65% in goats. We detected FMDV using molecular diagnostic tools such as RT-PCR and RT-LAMP and the phylogenetic analysis of the obtained sequences revealed the presence of FMDV serotype SAT 2, lineage II. Sequencing of the oropharyngeal fluid samples collected from African buffaloes revealed the presence of *Prevotela ruminicola, Spathidium amphoriforme, Moraxella bovoculi Onchocerca flexuosa*, *Eudiplodinium moggii, Metadinium medium* and *Verrucomicrobia bacterium* among other pathogens but no FMDV was detected in African buffaloes.

**Conclusions:**

We recommend further studies to focus on sampling more African buffaloes since the number sampled was statistically insignificant to conclusively exclude the presence or absence of FMDV in Eastern Rwanda buffaloes. The use of RT-PCR alongside RT-LAMP demonstrates that the latter can be adopted in endemic areas such as Rwanda to fill in the gaps in terms of molecular diagnostics. The identification of lineage II of SAT 2 in Rwanda for the first time shows that the categorised FMDV pools as previously established are not static over time.

**Supplementary Information:**

The online version contains supplementary material available at 10.1186/s12917-022-03430-1.

## Background

Foot-and-Mouth Disease Virus (FMDV) is a positive-sense RNA virus of the family of the picornaviridæ [1]. Based on the most variable part of the capsid, the VP1, FMDV is classified into seven serotypes (SAT1, SAT2, SAT3, O, A, C and Asia1) which are also subdivided into topotypes [2, 3]. Vaccination against one serotype does not confer protection against a different serotype and multivalent vaccines are often used [4–6]. Due to the constant change of this virus, a consistent molecular analysis is of paramount importance to improve the vaccines. Understanding the serological and molecular epidemiology of FMDV in Rwanda is very important because East Africa is considered to have the most divergent Foot-and-Mouth Disease (FMD) situation in the world [7].

In this study, we characterized the FMDV strains responsible for FMD outbreaks in Rwanda in 2017 and FMD seroprevalence in 2020. Molecular diagnostics were performed using Reverse Transcription Polymerase Chain Reaction (RT-PCR) and the pen-side Reverse Transcription Loop-Mediated Isothermal Amplification (RT-LAMP) chargeable and portable machine, the Axxin T8 isothermal instrument. This was complemented by sequencing the strains responsible for the 2017 FMD outbreaks in Eastern Rwanda. Moreover, we are presenting the serological situation of FMDV in large and small ruminants from the Eastern Province of Rwanda. These data will be crucial in policy guidance but also in studying risk factors. African buffaloes (*Syncerus caffer*) known to be natural wildlife reservoirs of FMDV were sampled [8, 9]. Though we did not isolate FMDV in African buffaloes, we identified other pathogens and commensals. To our knowledge, there is no published research on FMD seroprevalence in Rwanda and the latest available FMD molecular characterization results are from the 2001 FMD outbreak [10, 11].

## Results

### Seroprevalence

We collected samples from adult cattle and goats as follows: 823 cattle sera samples and 188 goat sera samples in 4 districts of the Eastern Province of Rwanda. In the 3ABC ELISA (ID Screen® FMD NSP Competition, ID-VET, Grabels, France) the overall prevalence was 9.36% (77/823, CI _95%:_ 7.5%-11.6%) in cattle and 2.65% (5/188, CI _95%:_ 0.9%-6.1%) in goats. The seroprevalence distribution in bovine was as follows; 8.6% (55/639, CI _95%:_ 6.5%-11.1%) in Bugesera and 11.96% (22/184, CI _95%:_ 7.6%-17.5%) in Nyagatare. In caprine, the seroprevalence was at 2.77% (4/144, CI _95%:_ 0.8%-7%) in Bugesera and 2.27% (1/44, CI _95%:_ 0.8%-7%) in Kayonza. The raw datasets presenting optical density to support these results are available at https://figshare.com/s/dbc28201eacecf5f2183.

### Reverse transcription polymerase chain reaction (RT-PCR)

**Fig. 1:**
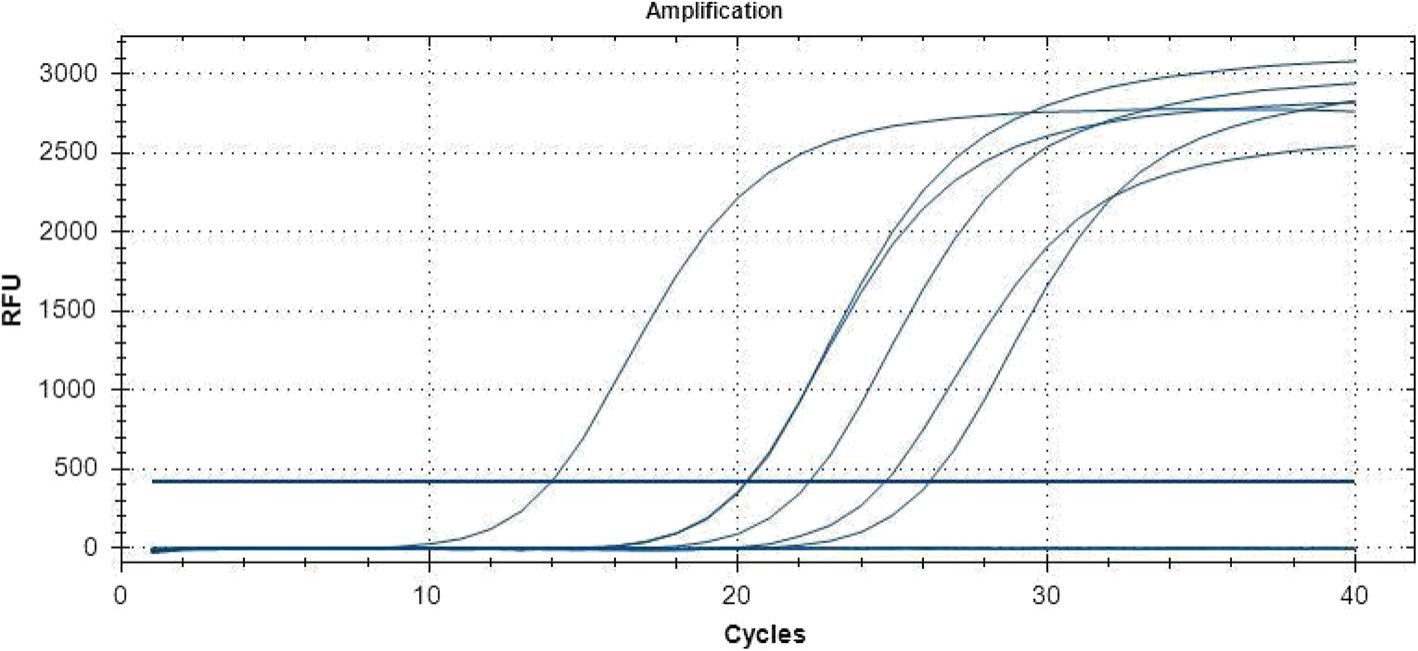
One-Step RT-PCR analysis. Amplification curves illustrating some of the select positive samples

**Fig. 2:**
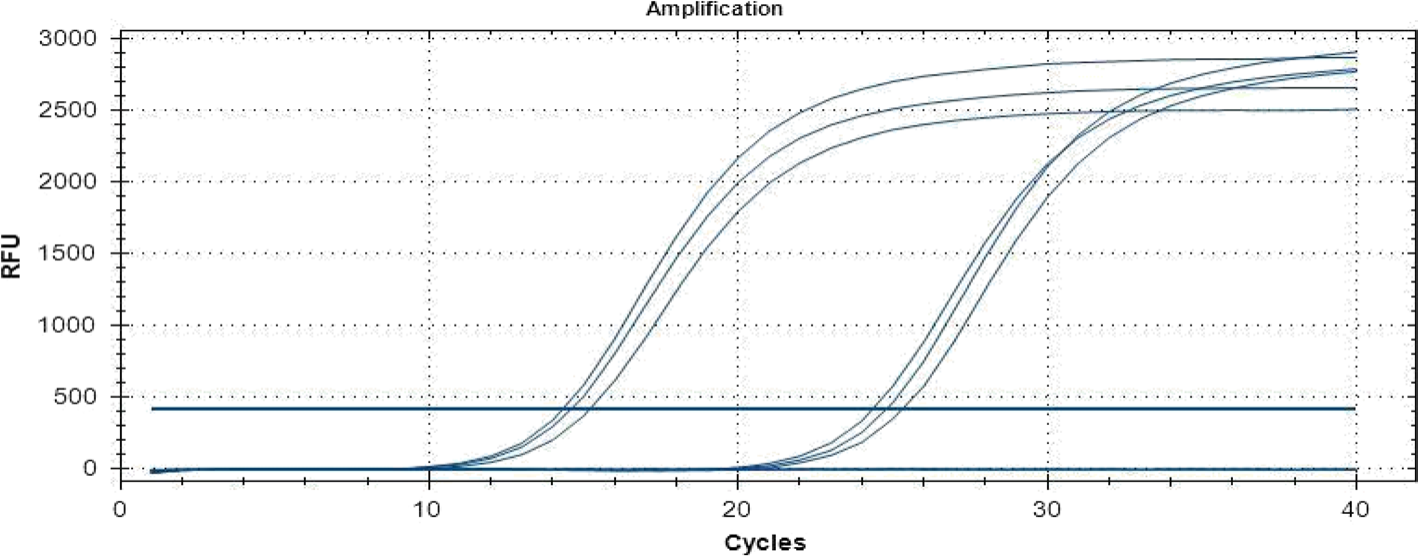
Early triplicates of sample 8 (from Gatsibo) and sample 26 (from Nyagatare)

The 2017 FMD outbreak samples from Eastern Rwanda were analysed by RT-PCR. The 1-step RT-PCR assays performed on the samples collected from the field samples demonstrated 6 out of 9 oropharyngeal (OP) samples positively identifying infection with the FMD virus. Select PCR positive samples are displayed in Fig. 1 and an increase in fluorescence intensity (Relative Fluorescence Units or RFU) was detected before the threshold of 32.0 cycles of amplification. Samples 8 and 26 representing Gatsibo and Nyagatare districts respectively were particularly chosen for further analysis. Figure 2 illustrates the triplicates results of select samples.

### Reverse transcription loop-mediated isothermal amplification (RT-LAMP)

**Fig. 3:**
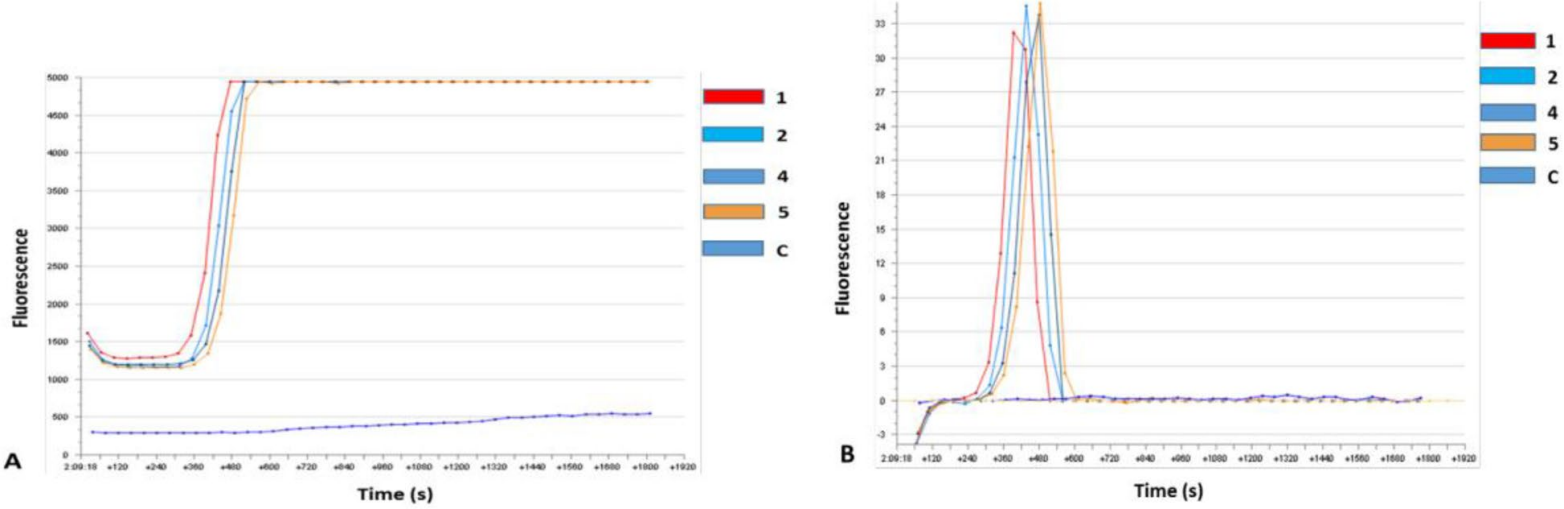
RT-LAMP results. **a**: trial time of fluorescence detection and **b**: the second derivative

**Fig. 4:**
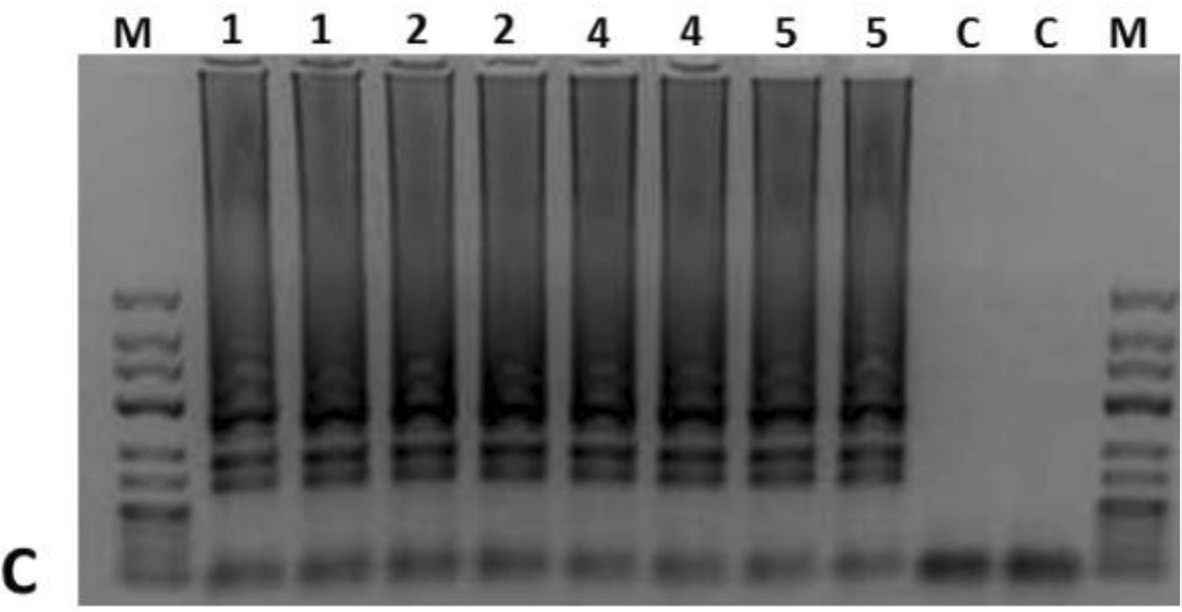
RT-LAMP cropped gel detection of sample duplicates. Lane M, DNA marker, 1–4, select positive samples

The RT-LAMP assay analysis revealed positive FMD detection consistent with results revealed by real-time PCR profile detection from the samples collected in the field. The time trial fluorescence graphs are represented in Fig. 3 and the gel-based detection in Fig. 4. The LAMP products gel displays a mixture of stem-loop DNA molecules of different sizes, a typical ladder pattern with many bands of different sizes. The successful amplification is indicated by the bands becoming darker in the later stages of the gel running. The obtained pattern is similar to the one obtained by Ding *et al*. to diagnose FMDV serotype C [12].

### Sequencing and phylogenetic inference

**Fig. 5:**
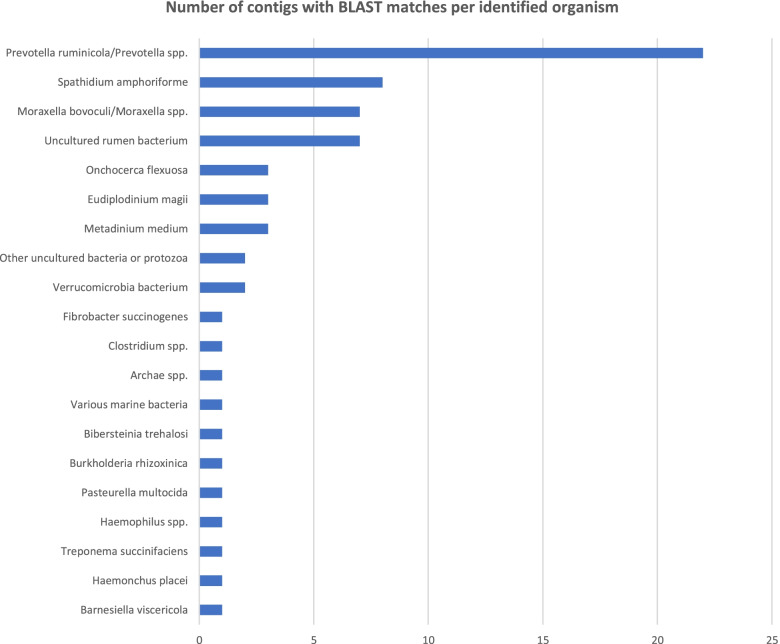
Sequenced contigs in A. buffaloes based on the top-scoring BLAST match for each contig

The oropharyngeal fluids (OPF) sampled from African buffaloes were subjected to non-targeted next-generation sequencing and the Raw data files are available at the Sequence Read Archive (SRA), NCBI and data information can be found at the BioProject (PRJNA865910). Based on BLAST searches of the contigs generated through sequencing (each > 400 bp in length), FMDV was not present in these OPF samples. However, many of the contigs showed significant alignment to other pathogens and commensals (Fig. 5), suggesting that African buffaloes may be reservoirs of other infectious diseases of interest that can infect domestic ungulates in Rwanda.

We identified *Bibersteinia trehalosi* [Accession No. SAMN30146891] in 1/7 African Buffaloes. This Gram-negative bacterium can cause systemic infection in sheep [13] and other species such as goats and cattle [14–16] and reared bison (*Bison bison*) [15, 17]. Yet, another isolated bacterium from 3/7 buffaloes was *Moraxella bovoculi* [Accession No. SAMN30146892 and SAMN30146888], responsible for infectious bovine kerato-conjunctivitis [18]. This implies the presence in the region of the most important vector of this bacterium, face flies (*Musca autumnalis*) [19]. *Mannheimia varigena* [Accession No. SAMN30146890] found in 1/7 buffaloes, is a bacterium that has a big range of host species [20] and is known for causing haemorrhagic septicaemia (HS) in cattle and water buffaloes. *M. varigena* has been reported to have a significant economic impact in Asia [21] and as the agent causing HS in African buffaloes [22].

*Haemophilus sp.* [Accession No. SAMN30146889] was also isolated in 1/7 of the sampled buffaloes. This blood-sucking nematode (hence the genus name) has been associated with anaemia, oedema and weight loss, and severe infections can result in death, particularly among young animals [23, 24]. Finding *Haemophilus* in Rwandan wildlife is consistent with its geographical location in a tropical and temperate region. Since this parasite was identified with NGS, clear differentiation was possible from *H. similis*, which is difficult to distinguish morphologically. In addition to the three pathogens and two parasites detected, various commensals were also identified, with the most predominant being *Prevotella ruminicola* [Accession No. SAMN30146884], followed by *Eudiplodinium maggii* [Accession No. SAMN30146886]. No previous study has reported commensals in African buffaloes (*Syncerus caffer caffer*) of Rwanda or other wild ruminants. The presence of commensals such as enterococci in African buffaloes might play a considerable role in antimicrobial resistance both in wild and domestic animals [25], future investigations need to explore it. The commensals and parasites identified in this study most likely originated from different water sources in the park as it has been suggested in other studies [26, 27]. These watering points may be shared with livestock and humans and an infection from one species to another is plausible. In addition, *Treponema sp.* [Accession: SAMN30146887] and *Bacteriodes sp.* [Accession: SAMN30146885] were also found.

FMDV whole genome was sequenced from OPF clinical samples collected from cattle and the sequences are available upon request to the corresponding author.

**Fig. 6:**
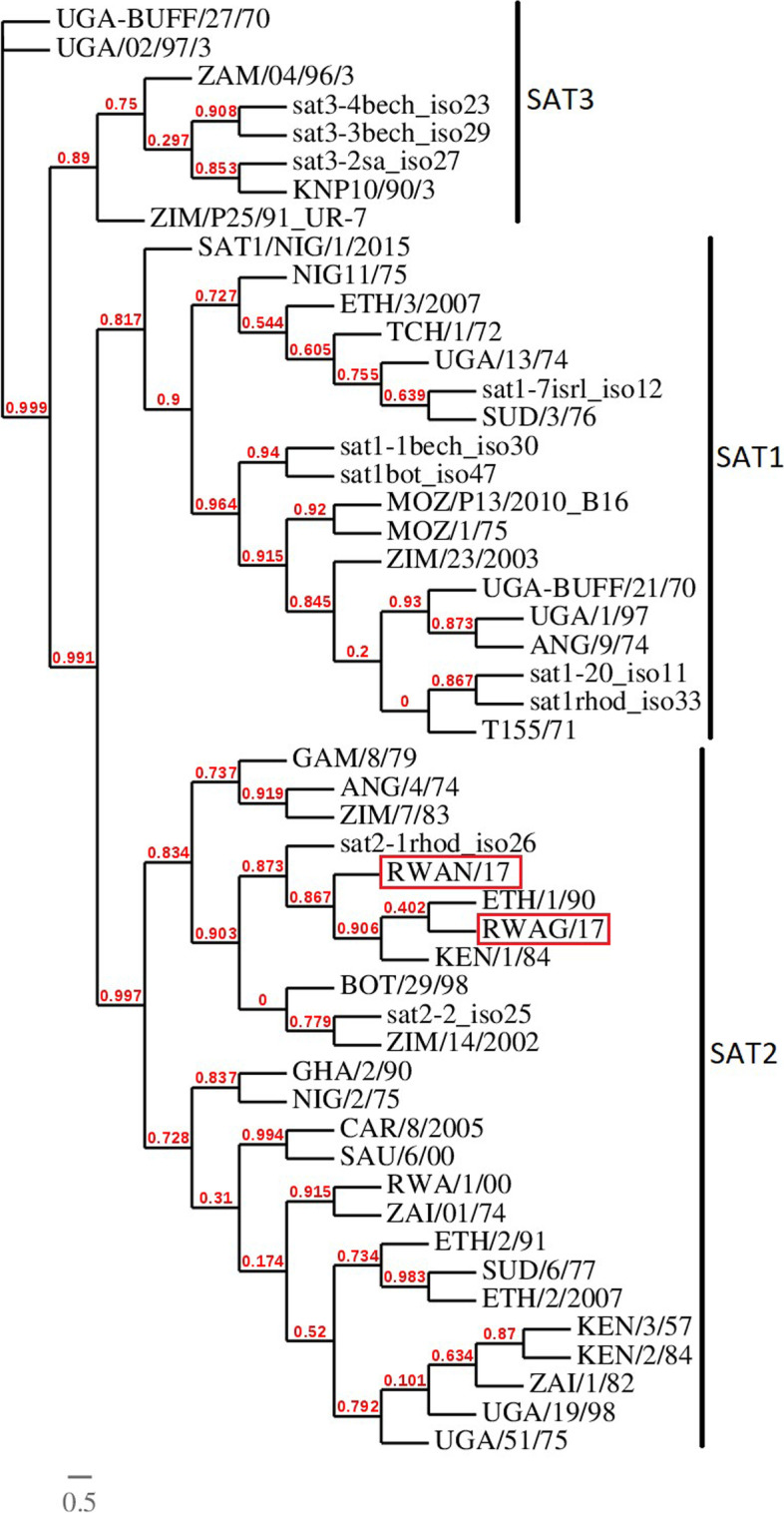
A Bayesian analysis showing different clades of SAT serotypes. Rwanda isolates are squared in red

Phylogenetic analyses of VP1 proteins from this study and prototypes available online at the national center for biotechnology information (www.ncbi.nlm.nih.gov) showed that the isolated viruses belonged to SAT 2. The generated tree (Fig. 6) shows a clade comprising a virus isolated from Zimbabwe in 1948 (sat2-1rhod_iso26) on one side. On the second branch of that clade, is this study’s isolate from Nyagatare district (Rwanda) in 2017 and another sub-branch comprising viruses from Kenya isolated in 1984 (KEN/1/84) and Ethiopia isolated in 1990 (ETH/1/90) and a virus isolated in Gatsibo district (Rwanda) in 2017. The evolutionary distance shows that the isolates from Rwanda have the smallest branch length followed by isolates KEN/1/84 and ETH/1/90 in East Africa.

## Discussion

### Seroprevalence

The detection of antibodies to NSP such as the highly conserved 3ABC is widely used to determine the FMDV seroprevalence [28]. In our 2020 study, we were not able to get information on seroprevalence for the study on FMD risk factors in Eastern Rwanda [29], therefore this study sets a baseline to get first-hand FMD seroepidemiological information in cattle and small ruminants in Eastern Rwanda. With 3ABC ELISA (Enzyme-Linked Immunosorbent Assay), antibodies against FMDV were detected at the sero prevalence of 9.36% and 2.65% in cattle and in goats respectively. The higher prevalence in cattle may be explained by the fact that viral replication is expected to be more luxuriant and more persisting in cattle than in small ruminants [30].

A sero-surveillance study in wild FMD-susceptible animals would be valuable to understand the role played by the wild animals. Moreover, we observed that in the visited farms in Bugesera there were many on-site water resources and fodder banks that decreased the risk of animals roaming outside the farms. This could have contributed to the lower seroprevalence in Bugesera as compared to Nyagatare.

### Cattle samples

Regions in Eastern Rwanda, at the border between Uganda and Tanzania, are at risk of transnational or transboundary circulation of the FMD virus. We characterised the strains responsible for the 2017 FMD outbreak as SAT 2. This serotype appears to be the most predominant serotype in Sub-Saharan Africa [31, 32]. It is probably possible that the uncontrolled transboundary livestock movements in this region could be contributing to the presence of the current circulating FMDV strains that are identified in this area.

### Reverse Transcription Polymerase Chain Reaction (RT-PCR)

Amplification using specific primers targeting FMDV SAT2 serotype revealed the presence of serotype SAT 2 in the OP samples. Although FMDV SAT 2 appears to be the most predominant in East Africa in general [33], the majority of outbreaks in Kenya have been caused by serotypes O and SAT2 [34], in Uganda by serotype O and SAT 2 while in Rwanda the last reported outbreak in 2004 belonged to serotype O [35]. The current finding shows that that SAT2 was responsible for the 2017 FMD outbreak. This calls for the need to incorporate SAT 2 virus strains in vaccines that may be used in Rwanda areas if a regionally coordinated vaccination campaign is to be carried out.

### Reverse transcription loop-mediated isothermal amplification (RT-LAMP)

This method relies on auto-cycling strand displacement DNA synthesis that is performed by DNA polymerase with high strand displacement activity and a set of two specially designed inner and two outer primers [36].

FMDV constant molecular diagnostics allow a better understanding of circulating strains for a smarter vaccination. In this study, we have confirmed what other studies have established that LAMP technology can be an alternative to traditional PCR since it is very portable and can yield reliable results. Our LAMP results have shown that SAT 2 serotype was responsible for the 2017 outbreak, this in concordance with PCR results. This assay has been previously used in Southern Africa successfully on FMDV SATs serotypes [37].

The LAMP technology can be very useful for a point-of-care detection during an active outbreak to detect FMDV while sampling is still going on. The Axxin T8 being a portable device with batteries make it suitable to be used in mobile laboratories and since RT-LAMP skips the cDNA synthesis step and the extraction step for some samples such as blood [38, 39], it is much more fit for Low-and-Middle Income Countries and decreases the risk of contamination [40]. RT-LAMP has proved to detect positive samples and within 30 min we were able to molecularly diagnose FMDV which would take a much longer time with laborious manipulations when diagnosing with RT-PCR. Also, RT-LAMP is better for blood samples because it is not hindered by inhibitory substances [40]. Recently, Mahapatra *et al.* have demonstrated that LAMP’s concordance with RT-qPCR reaches 100% [41]. This pen-side, rapid and cost-effective technology can be of great importance to quickly identify an outbreak and quarantine the area for further investigation. The democratisation of such technologies would fill the gap of lack of proper knowledge on circulating strains in Eastern Rwanda and other countries with similar challenges.

### Sequencing and phylogenetic inference

We did not detect FMDV in the seven samples of the African buffaloes randomly selected from the Nyamirama area inside the Akagera National Park in Rwanda by total RNA sequencing without prior amplification and *De Novo* sequence assembly. In South Africa, a pattern of infection from wildlife animals considered as natural reservoirs to livestock has been established, particularly African buffaloes (*Syncerus caffer*) [42, 43]. However, in Uganda Dhikusooka *et al.* were not able to confirm this in cases of SAT 3 and SAT 1 [44]. In South Africa, physical separation consisting of electro-fencing of parks and movement restriction combined with effective vaccination campaigns [45, 46] have assisted in reducing outbreaks from cross-infection and FMD control. However, in East Africa, few studies have been carried out on the circulating strains of FMDV, which constitutes a knowledge gap that prevents any conclusions that such measures have been similarly effective in this region. In 1979, SAT 3 was detected in African buffaloes in Queen Elizabeth National Park and in 2013 a healthy long-horned calf that grazed near this park [44]. But even then, the finding could not prove the possibility of cross infection between buffaloes and cattle that intermingled in Uganda, an area that is very close to Rwanda.

Several reports have demonstrated that African buffaloes play an important role in the epidemiology of the SAT serotypes of FMDV [47–49]. In the present study, the sampled buffaloes had been enclosed inside the park and separated from farm access with an electrical fence since 2013 [50]. Although there is a need for a greater number of buffalo samples to be analysed, the results of our study suggest that the series of outbreaks observed in Eastern Rwanda between 2000 and 2017 may be arising from different sources, probably from transboundary and intra-national livestock movements as well as proximity with unvaccinated small ruminants although this is not proved yet. These factors would contribute to virus persistence in the area because FMDV can be recovered in small ruminants up to 9 months post-infection [51].

Our results suggest that the isolated virus in Rwanda may have evolved and circulated from Zimbabwe to East Africa (Kenya) and spread in two branches Northward to Ethiopia and Southward to Rwanda. The 2017 sequences are quite different from the sequences previously characterized in Rwanda (isolate RWA/1/00) and in the neighbouring Democratic Republic of Congo (isolate ZAI/01/74). This shows the complexity of circulating strains in this region and considering that many outbreaks are unnoticed and or unreported, more strains might be circulating.

## Conclusion

While there were no FMDV pathogens isolated in African buffaloes, the whole genome sequencing revealed the presence of other pathogens that could also cross infect cattle. The plethora of pathogens identified from the buffalo gut is a signal to the livestock health department to establish whether some of the challenges they are facing do arise from the interactions that exist between domestic animals and wildlife at this interface.

The 2017 FMD outbreak in Eastern Rwanda was caused by SAT 2 serotypes and the VP1 phylogenetic analysis of the 2017 sequences showed for the first-time evidence of the presence of SAT 2 lineage II in Rwanda. This finding highlights the probable incursion of new FMD virus strains in the country. However, it was not possible to establish whether the origin was from wildlife or domesticated animal that are involved in the intercountry trade markets.

It is noteworthy to recognise that the RT-LAMP diagnostic tools that was used in this area can be a reliable rapid, and cost-effective alternative method for field detection of FMD in this country. This is because this technology does not require high molecular biology skills to be operated and thus can be promoted for use by a bigger number of field animal health workers.

## Methods

### Sampling techniques and source of animals

Seven (*n* = 7) healthy-looking mature African buffaloes were randomly immobilized inside the park in Nyamirama area. They were darted with a tranquiliser from a 4 × 4 vehicle using a JM Special dart gun with a 13 mm barrel loaded with a 2 mL Pneu-Dart and then injected with a 3.8 cm barbed needle using a spring-loaded pole syringe (Dan-Inject). The animals were darted in the hindquarters with 8 mg etorphine and 48 mg azaperone [52], that brought them down in sternal recumbence. After falling down, their faces were covered up to the ears with a cloth tissue, to avoid early wake-up. We then gave them 150 mg of Ketamine to increase muscle relaxation and jaw-bone movements. After going down, they received an additional 200 mg ketamine IV and 2 mg etorphine with 40 mg azaperone IM to quicken early waking up. After sample collection, these buffaloes were roused with up to 20 mg diprenorphine and 100 mg naltrexone given intravenously [52, 53]. Throughout the sample collection, we monitored the buffaloes’ breathing and no animal needed a respirator stimulant or partial antagonism. We collected OPF and scraps using a probang cup and transferred the samples to sterile tubes containing an equal amount of transport media. We inserted a probang cup in the OP tract and vigorously passed it with back-and-forth movements at least 5–10 times between the first portion of the oesophagus and the back of the pharynx. Tubes had equal amounts of glycerol and 0.04 M phosphate buffer (pH 7.2–7.6) containing 1 × antibiotic–antifungal mixture (Thermo-Fisher Scientific, Johannesburg, South Africa) [54, 55]. Between OPF collection from one animal to the next using the OIE three-bucket washing system slightly modified [54, 55]. We washed the probang cup in a bucket containing 0.3% citric acid, rinsed it in another bucket with water and lastly disinfected the cup in Phosphate-buffered Saline. The sample tubes were topped up to contain an equivalent volume of transport medium to that of the sample.

Following the described methodology above, in July 2017, we collected from crossbred (Ankole x Jersey) cattle (*n* = 9) OPF and scraps from cattle in the Gatsibo and Nyagatare districts during the 2017 FMD outbreak. Samples were collected from animals presenting clinical signs such as blisters on the mouth or the foot. Collecting samples from animals, as described later in this section, was done in compliance with guidelines provided in the USDA’s Foreign Animal Disease Investigation Manual [56] and approved by the Rwanda Agriculture Board. We transported samples in cooler boxes on ice from the field to the Virology Laboratory of the Rwanda Agriculture and Animal Resources Development Board located in Kigali, Rwanda and stored them at − 80 °C until further processing. The animals used in this study were African buffaloes (*Syncerus caffer*) from the Akagera National Park, healthy-looking Cattle and Goats from individual farms in Eastern Rwanda and Cattle from one infected farm in Eastern Rwanda.

### Serological analyses

Sera samples were randomly collected from three districts of the Eastern province during surveillance. Samples were stored at -20 °C for less than one week before analysis. We used the ID Screen® FMD NSP Competition Kit (ID.Vet, Grabels, France) according to the manufacturer’s instructions to detect the non-structural protein 3ABC in serum. The test was applied to samples from cattle (*Bos taurus*) (*n* = 823) and goats (*Capra aegagrus hircus*) (*n* = 188) collected in the Eastern Province of Rwanda.

### Reverse transcription polymerase chain reaction

#### RNA extraction and cDNA synthesis

RNA was manually extracted using the PureLink Viral RNA/DNA Kit according to manufacturer’s instructions. We added 200 μL of OPF sample to a Kit Master Mix (proteinase K, lysis buffer, carrier RNA, and 100% ethanol). We performed a two-step wash using a wash buffer solution, and eluate in 60 μL nuclease-free water was collected and transferred to 1.5 ml tubes. We treated the eluate with DNase using the Turbo DNA-free Kit and kit manual, to remove host genomic DNA. Thereafter, we collected 50 μL of DNA-free eluate and transferred it to 1.5 mL tubes for downstream analyses. We quantified the nucleic material in the collected solutions using the Quantus™ Fluorometer. The SuperScript VILO cDNA Synthesis Kit and kit manual were used for the conversion of RNA extracted manually, and 20 μL from that extracted using the KingFisher Duo machine, to cDNA. We added 10 μL of RNA eluate to a master mix containing 5X VILO reaction mix, 10X SuperScript enzyme mix and nuclease-free water. Complementary DNA synthesis was achieved at 42 °C for 60 min. Thereafter, we cleaned up the cDNA using the Macherey–Nagel™ Nucleospin Gel and PCR Clean-up Kit and kit manual. Finally, we added 50 μl cDNA to Buffer NE, Buffer NT1, and Buffer NT3 following the methodology detailed in the kit manual.

### RT-PCR

#### Primers

We adopted primers and probes of the recommended protocol by the World Reference Laboratory for FMD (WRLFMD) for amplification (conventional PCR) and for VP1 sequencing. These primers are designed to detect all the seven serotypes of FMDV and do not amplify other viruses including viruses similar to FMDV responsible for vesicular diseases [57]. The rRT-PCR primers used in this study are described in Table 1.

**Table 1:** List of primers used for a rRT-PCR for the detection of FMDV in this study [58]

Primer/probe	Oligo name	**Primer sequence 5’-3’**	**Genome direction**	**Working concentration**
**Forward primer**	3DF	ACTGGGTTTTACAAACCTGTGA	Forward	10 pmol/μl
**Reverse primer**	3DR	GCGAGTCCTGCCACGGA	Reverse	10 pmol/μl
**Taqman probe**	3DP	TCCTTTGCACGCCGTGGGAC	Probe	5 pmol/μl

Considering the primers we used for amplifying SAT2 VP1 portions, Table 2 displays the primers that are, according to the WRLFMD, appropriate to be used for sequencing.

**Table 2:** List of oligonucleotide primers used for SAT2 VP1 sequencing

Primer name	Sequence (5’ – 3’)	Genome direction	Gene
SAT2-D	GGTGCGCCGTTGGGTTGCCA	Reverse	VP1
SAT2–1D209aF	CCACTTACTACTTTTGTGACCTTGA	Forward	
SAT2–1D209bF	CCACCTACTACTTTTGTGACCTTGA		
SAT2–1D209cF	CCACCTACTATTTCTGTGACCTGGA		
SAT2–1D209dF	CCACGTACTACTTCTGTGACCTGGA		

#### Amplification

The OP samples from the 2017 FMD outbreak were analysed using a multiplex one-step RT-PCR assay. The assay was performed using the TaqMan® Fast Virus 1-Step Kit (Life Technologies) and kit manual. We added 2 μL of eluate extracted in the manual extraction section to a Master Mix containing nuclease-free water, 4X TaqMan buffer, primers and TaqMan probe. The thermal cycler (BioRad, Hercules, California) was programmed as follows; reverse transcription at 50 °C for 5 min; polymerase activation and DNA denaturation at 95 °C for 20 s; two-step amplification for 40 cycles with denaturation at 95 °C for 3 s; annealing and plate read at 60 °C for 30 s.

We analysed the PCR reaction to observe any positive FMD identification from the OPF samples collected and to compare the results obtained to those from the positive high and low standard controls. Reactions were considered positive if the amplification was detected before 32.0 cycles [59].

Before sequencing, we removed the primers and deoxynucleoside by enzymatically adding the ExoSAP-IT PCR Product Cleanup Reagent (0.5 μl of Exo and 2 μl of SAP) to 10 μl of amplicons. The mix was incubated at 37 °C for 15 min and later 85 °C for 15 to activate Exo and SAP respectively, lastly, the product was held at 4 °C.

### Reverse transcription loop-mediated isothermal amplification (RT-LAMP)

Amplification was performed on the Axxin T8–ISO instrument at 65 °C for 30 min. The assays were performed using the Isothermal Master Mix. Briefly; 6.5 μL cDNA was added to an FMD LAMP primer mix and isothermal master mix. The RT-LAMP assay we used was not specific for any serotype, it could only assess if a sample is positive or negative. Amplified products were detected both in real-time and by running an electrophoresis-based gel. The 2% agarose gel bands were read under U.V. light after staining with ethidium bromide. Primers and probes were used according to the manufacturer’s instructions (Tokabio (Pty) Ltd, Johannesburg, South Africa), they are available upon request from the corresponding author.

### Sequencing and Phylogenetic inference

We conducted the Whole Genome sequencing of the African buffaloes’ samples without necessarily running a genome-specific PCR amplification. We processed the buffalo samples with the PureLink® Viral RNA/DNA Mini Kit (Invitrogen, Carlsbad, CA) and treated them with the Turbo DNA-free™ Kit (Ambion, TX, USA) to remove any residual host DNA. We then used the Superscript VILO cDNA Synthesis Kit (Invitrogen, Carlsbad, CA) to generate cDNA from the isolated RNA.

NGS libraries were prepared from the samples with the Ion Xpress™ Plus Fragment Library Builder Kit on the AB Library Builder system (Life Technologies). Each sample was uniquely barcoded during library preparation using the Ion Xpress Barcode Adapters 1–16 Kit. We performed template preparation with the Ion PGM Template OT2 200 Kit and the OneTouch 2 instrument, and the samples were sequenced on the Ion PGM next-generation sequencer using the Ion PGM Sequencing 200 Kit. All reagents and instruments were purchased from Thermo Fisher Scientific, Johannesburg, South Africa. Data were analysed with CLC Genomics Workbench Software v.10 (Qiagen Bioinformatics, Redwood City, CA, USA) and the NCBI database with the BLAST tool (https://blast.ncbi.nlm.nih.gov/Blast.cgi). Alongside the sequences produced by this study, we retrieved FMDV SAT prototype sequences from the National Center for Biotechnology Information (NCBI).

Using the NGphylogeny.fr platform, select SAT isolates were aligned with MAFFT version 7 [60] and cleaned by Gblocks [61, 62], using the MrBayes using the GTR model [63] embedded software trees were constructed and visualised by Newick display [64].

## Supplementary Information

**Additional file 1.** Retrieved sequences used in this study.

**Additional file 2.** FMD serological NSP results from cattle and goats in Eastern Rwanda.

## Data Availability

The serological datasets generated in this study are available in the figshare repository, https://figshare.com/s/dbc28201eacecf5f2183 or using the following https://doi.org/10.6084/m9.figshare.14890977. Raw sequences data supporting the molecular analysis are not yet public due to ongoing research by TokaBio with commercial interests, data are however available from the authors upon reasonable request and with permission of TokaBio (Pty) Ltd. We provided details of the retrieved sequences and phylogenetic branch length in a public repository (https://figshare.com/articles/dataset/Select_FMDV_SAT_prototype_sequences_and_Bayesian_branch_length/16679863). The sequencing dataset generated in this study has been deposited to NCBI Short Read Archive (SRA) under a BioProject with accession number PRJNA865910.

## Abbreviations

FMDV: Foot-and-Mouth Disease Virus
FMD: Foot-and-Mouth Disease
SAT: Southern African Territories
RT-PCR: Reverse Transcription Polymerase Chain Reaction
RT-LAMP: Reverse Transcription Loop-Mediated Isothermal Amplification
CI: Confidence Interval
OP: Oro-pharyngeal
OPF: Oro-pharyngeal fluids
RFU: Relative Fluorescence Units
OIE: Organisation Internationale des Epizooties
NSP: Non-structural proteins
ELISA: Enzyme-Linked Immunosorbent Assay
ANP: Akagera National Park
NCBI: National Centre for Biotechnology Information
WRLFMD: World Reference Laboratory for Foot and Mouth Disease

## References

[1] Domingo E, Baranowski E, Escarmis C, Sobrino F (2002). Foot and mouth disease viruses. Comp Immunol Microbiol Infect Dis.

[2] Jamal SM, Belsham GJ (2013). Foot-and-mouth disease: past, present and future. Vet Res.

[3] Ayelet G, Mahapatra M, Gelaye E, Egziabher BG, Rufeal T, Sahle M (2009). Genetic characterization of foot-and-mouth disease viruses, Ethiopia, 1981–2007. Emerg Infect Dis.

[4] Brooksby JB (1982). Portraits of viruses: foot-and-mouth disease virus. Intervirology.

[5] Cartwright B, Chapman WG, Sharpe RT (1982). Stimulation by heterotypic antigens of foot-and-mouth disease virus antibodies in vaccinated cattle. Res Vet Sci.

[6] Mattion N, König G, Seki C, Smitsaart E, Maradei E, Robiolo B (2004). Reintroduction of foot-and-mouth disease in Argentina: characterisation of the isolates and development of tools for the control and eradication of the disease. Vaccine.

[7] Rweyemamu M, Roeder P, Mackay D, Sumption K, Brownlie J, Leforban Y (2008). Epidemiological patterns of foot-and-mouth disease worldwide. Transbound Emerg Dis.

[8] Hedger RS (1972). Foot-and-mouth disease and the African buffalo (Syncerus caffer). J Comp Pathol..

[9] Vosloo W, Boshoff K, Dwarka R, Bastos A (2002). The possible role that buffalo played in the recent outbreaks of foot-and-mouth disease in South Africa. Ann N Y Acad Sci.

[10] National Center for Biotechnology Information. Foot-and-mouth disease virus - type SAT 2 isolate RWA/02/01 leader proteinase mRNA, partial cds. Bethesda (MD): National Library of Medicine (US), National Center for Biotechnology Information. 2005. Available from: https://www.ncbi.nlm.nih.gov/nuccore/AY878708.1. [Cited 2022 Jun 8].

[11] National Center for Biotechnology Information. Foot-and-mouth disease virus SAT 2 isolate RWA/2/01 3C proteinase mRNA, partial cds. Bethesda (MD): National Library of Medicine (US), National Center for Biotechnology Information. 2005. Available from: https://www.ncbi.nlm.nih.gov/nuccore/AY884139.1

[12] Ding Y, Zhou J, Ma L, Qi Y, Wei G, Zhang J (2014). A reverse transcription loop-mediated isothermal amplification assay to rapidly diagnose foot-and-mouth disease virus C. J Vet Sci.

[13] Blackall PJ, Bojesen AM, Christensen H, Bisgaard M (2007). Reclassification of [Pasteurella] trehalosi as Bibersteinia trehalosi gen nov, comb nov. Int J Syst Evol Microbiol.

[14] Wood ME, Fox KA, Jennings-gaines J, Killion HJ, Amundson S (2017). How Respiratory Pathogens Contribute to Lamb Mortality in a Poorly Performing Bighorn Sheep ( Ovis canadensis ) Herd. J Wildl Dis.

[15] Hanthorn CJ. Pathogenicity of Bibersteinia trehalosi in bovine calves. Iowa State University; 2014. Available from: http://lib.dr.iastate.edu/cgi/viewcontent.cgi?article=4981&context=etd

[16] Scott PR (2011). Treatment and Control of Respiratory Disease in Sheep. Veterinary Clinics of NA: Food Animal Practice.

[17] Ward ACS, Dyer NW, Fenwick BW (1999). Pasteurellaceae isolated from tonsillar samples of commercially- reared American Bison ( Bison bison ). Can J Vet Res.

[18] Dickey AM, Loy JD, Bono JL, Smith TPL, Apley MD, Lubbers BV (2016). Large genomic differences between Moraxella bovoculi isolates acquired from the eyes of cattle with infectious bovine keratoconjunctivitis versus the deep nasopharynx of asymptomatic cattle. Vet Res.

[19] Brown MH, Brightman AH, Fenwick BW, Rider MA (1998). Infectious bovine keratoconjunctivitis: a review. J Vet Intern Med.

[20] Wilkie IW, Harper M, Boyce JD, Adler B. Pasteurella multocida: Diseases and Pathogenesis. In: Current Topics in Microbiology and Immunology. Clayton, Australia; 2012. p. 1–22. Available from: https://books.google.com/books?id=_DDwCqx6wpcC&printsec=frontcover&dq=unwritten+rules+of+phd+research&hl=&cd=1&source=gbs_api%255Cnpapers2://publication/uuid/48967E01-55F9-4397-B941-310D9C5405FA%255Cn 10.1007/82_2012_216 22643916

[21] Shivachandra SB, Viswas KN, Kumar AA (2011). A review of hemorrhagic septicemia in cattle and buffalo. Anim Heal Res Rev.

[22] The Center for Food Security and Public Health. Hemorrhagic Septicemia. 2019. p. 1–6. Available from: http://www.cfsph.iastate.edu/Factsheets/pdfs/hemorrhagic_septicemia.pdf. [Cited 2019 Sep 23].

[23] Gennari SM, Abdalla AL, Vitti DMSS, Meirelles CF, Lopes RS, Bressan MCRV. Haemonchus placei in calves : effects of dietary protein and multiple experimental infection on worm establishment and pathogenesis. 1995;59(2):119–26. Available from: https://www.sciencedirect.com/science/article/pii/030440179400741T 10.1016/0304-4017(94)00741-t 7483235

[24] Arzoun IH, Hussein HS, Hussein MF. Some of the clinico-pathological features in experimentally-induced Hae- monchus longistipes infection in Sudanese one-humped camels ( Camelus dromedarius ). Animals Six camels were purchased from a local market in Omdurman , Khartoum Parasitological meth. Vet Parasitol. 1984;14(1):43–53. Available from: https://www.sciencedirect.com/science/article/pii/0304401784901328

[25] Shin E, Mduma S, Keyyu J, Fyumagwa R, Lee Y (2017). An investigation of Enterococcus species isolated from the African Buffalo (Syncerus caffer) in Serengeti National Park Tanzania. Microbes Environ.

[26] VanderWaal KL, Atwill ER, Isbell LA, McCowan B (2014). Quantifying microbe transmission networks for wild and domestic ungulates in Kenya. Biol Conserv.

[27] VanderWaal K, Omondi GP, Obanda V (2014). Mixed-host aggregations and helminth parasite sharing in an East African wildlife-livestock system. Vet Parasitol.

[28] Sørensen KJ, Hansen CM, Madsen ES, Madsen KG (1998). Blocking ELISAs using the FMDV non-structural proteins 3D, 3AB, and 3ABC produced in the baculovirus expression system. Vet Q.

[29] Udahemuka JC, Aboge GO, Obiero GO, Lebea PJ, Onono JO, Paone M (2020). Risk factors for the incursion, spread and persistence of the foot and mouth disease virus in Eastern Rwanda. BMC Vet Res.

[30] Rout M, Senapati MR, Mohapatra JK, Dash BB, Sanyal A, Pattnaik B (2014). Serosurveillance of foot-and-mouth disease in sheep and goat population of India. Prev Vet Med.

[31] Doel T (2003). FMD vaccines. Virus Res.

[32] Kotecha A, Seago J, Scott K, Burman A, Loureiro S, Ren J (2015). Structure-based energetics of protein interfaces guides foot-and-mouth disease virus vaccine design. Nat Struct Mol Biol.

[33] Kerfua SD, Shirima G, Kusiluka L, Ayebazibwe C, Mwebe R, Cleaveland S (2018). Spatial and temporal distribution of foot-and-mouth disease in four districts situated along the Uganda – Tanzania border : implications for cross-border efforts in disease control. Onderstepoort J Vet Res.

[34] Sangula AK, Belsham GJ, Muwanika VB, Heller R, Balinda SN, Masembe C, Siegismund HR (2010). Evolutionary analysis of foot-and-mouth disease virus serotype SAT 1 isolates from east Africa suggests two independent introductions from southern Africa. BMC Evol Biol..

[35] WRLFMD. Rwanda: (Kayonza) cattle, serotype pending, OIE. Surrey: Pirbright Institute; 2020.

[36] Notomi T (2000). Loop-mediated isothermal amplification of DNA. Nucleic Acids Res.

[37] Bhoora RV, Lebea PJ, Maree FF (2014). Towards the development of a pen-side diagnostics strategy for controlling foot and mouth disease virus within the control zones of the southern african development community (SADC) member states - phase 1.

[38] Kaneko H, Kawana T, Fukushima E, Suzutani T (2007). Tolerance of loop-mediated isothermal amplification to a culture medium and biological substances. J Biochem Biophys Methods.

[39] Poon LL, Wong BW, Ma EH, Chan KH, Chow LM, Abeyewickreme W (2006). Sensitive and inexpensive molecular test for falciparum malaria: detecting plasmodium falciparum DNA directly from heat-treated blood by loop-mediated isothermal amplification. Clin Chem.

[40] Blomström A-L, Hakhverdyan M, Reid SM, Dukes JP, King DP, Belák S (2008). A one-step reverse transcriptase loop-mediated isothermal amplification assay for simple and rapid detection of swine vesicular disease virus. J Virol Methods.

[41] Mahapatra, Howson, Fowler, Batten, Flannery, Selvaraj, et al. Rapid Detection of Peste des Petits Ruminants Virus (PPRV) Nucleic Acid Using a Novel Low-Cost Reverse Transcription Loop-Mediated Isothermal Amplification (RT-LAMP) Assay for Future Use in Nascent PPR Eradication Programme. Viruses. 2019;11(8):699. Available from: https://www.mdpi.com/1999-4915/11/8/699 10.3390/v11080699 PMC6723471 31370329

[42] Hedger RS. Foot-and-Mouth Disease with particular reference to the African buffalo (Syncerus caffer). Wildl Dis. 1976;235–44.

[43] Thomson GR, Vosloo W, Esterhuysen JJ, Bengis RG (1992). Maintenance of foot and mouth disease viruses in buffalo in Southern Africa. Rev Sci Tech.

[44] Dhikusooka MT, Tjørnehøj K, Ayebazibwe C, Namatovu A, Ruhweza S, Siegismund HR (2015). Foot-and-Mouth Disease Virus Serotype SAT 3 in Long-Horned Ankole Calf. Uganda Emerg Infect Dis.

[45] Thomson G (1995). Overview of foot and mouth disease in southern Africa. Rev Sci Tech.

[46] Thomson G, Vosloo W, Bastos AD. Foot and Mouth Disease in Southern Africa: Re-evaluation of the approach to control. In: 1th symposium on Diagnosis and Control of Transboundary Infectious Diseases in Southern Africa. Utrecht: Tropical Animal Health and Production, 11th Symposium; 2000. Available from: https://www.researchgate.net/publication/263814752_Foot-and-mouth_disease_in_southern_Africa_re-evaluation_of_the_approach_to_control

[47] Anderson EC, Doughty WJ, Anderson J, Paling R (1979). The pathogenesis of foot-and-mouth disease in the African buffalo (Syncerus caffer) and the role of this species in the epidemiology of the disease in Kenya. J Comp Pathol.

[48] Di Nardo A, Libeau G, Chardonnet B, Chardonnet P, Kock RA, Parekh K (2015). Serological profile of foot-and-mouth disease in wildlife populations of West and Central Africa with special reference to Syncerus caffer subspecies. Vet Res.

[49] Ayebazibwe C, Mwiine FN, Tjørnehøj K, Balinda SN, Muwanika VB, Ademun Okurut AR, Belsham GJ, Normann P, Siegismund HR, Alexandersen S (2010). The role of African buffalos (syncerus caffer) in the maintenance of foot-and-mouth disease in Uganda. BMC Vet Res..

[50] Bariyanga JD, Wronski T, Plath M, Apio A (2016). Effectiveness of electro-fencing for restricting the ranging behaviour of wildlife: a case study in the degazetted parts of Akagera National Park. African Zool.

[51] Moonen P, Schrijver R (2000). Carriers of foot-and-mouth disease virus: a review. Vet Q.

[52] Hoffman LC, Hildebrandt WR, Leslie AJ (2018). Chemical composition of African Savanna Buffalo (Syncerus caffer) meat. African J Wildl Res.

[53] Clarke C, Cooper D, Goosen WJ, McFadyen R, Warren RM, van Helden PD (2018). Antigen-specific interferon-gamma release is decreased following the single intradermal comparative cervical skin test in African buffaloes (Syncerus caffer). Vet Immunol Immunopathol.

[54] Paton D, King D. Diagnostic and Sampling procedures for FMD Diagnostic windows. Available from: http://www.fao.org/ag/againfo/commissions/docs/training/material/Diagnostic_sampling_procedures/Diagnostic_sampling_procedures.pdf. [cited 2019 Aug 26].

[55] OIE (2009). Foot and Mouth Disease. OIE Terrestrial Manual 2009, Version adopted by the World Assembly of Delegates.

[56] USDA. FOREIGN ANIMAL DISEASE (FAD) INVESTIGATION MANUAL Foreign Animal Disease Preparedness & Response Plan Surveillance , Preparedness , and Response Services. 2017.

[57] Knowles NJ, Wadsworth J, King DP (2017). VP1 sequencing protocol for foot and mouth disease virus molecular epidemiology. Rev Sci Tech.

[58] Callahan JD, Brown F, Osorio FA, Sur JH, Kramer E, Long GW (2002). Use of a portable real-time reverse transcriptase-polymerase chain reaction assay for rapid detection of foot-and-mouth disease virus. J Am Vet Med Assoc..

[59] Shaw AE, Reid SM, Ebert K, Hutchings GH, Ferris NP, King DP (2007). Implementation of a one-step real-time RT-PCR protocol for diagnosis of foot-and-mouth disease. J Virol Method.

[60] Katoh K, Standley DM (2013). MAFFT multiple sequence alignment software version 7: improvements in performance and usability. Mol Biol Evol.

[61] Castresana J (2000). Selection of conserved blocks from multiple alignments for their use in phylogenetic analysis. Mol Biol Evol.

[62] Talavera G, Castresana J (2007). Improvement of phylogenies after removing divergent and ambiguously aligned blocks from protein sequence alignments. Kjer K, Page R, Sullivan J, editors. Syst Biol.

[63] Huelsenbeck JP, Ronquist F (2001). MRBAYES: Bayesian inference of phylogenetic trees. Bioinformatics.

[64] Lemoine F, Correia D, Lefort V, Doppelt-Azeroual O, Mareuil F, Cohen-Boulakia S (2019). NGPhylogeny.fr: new generation phylogenetic services for non-specialists. Nucleic Acids Res.

