# Supplementary material for: Investigation of foot and mouth disease virus and other animal pathogens in cattle, buffaloes and goats at the interface with Akagera National Park 2017 – 2020
Source: BMC Vet Res. 2022 Sep 16;18:349. doi: 10.1186/s12917-022-03430-1 (PMC9479285; doi:10.1186/s12917-022-03430-1)
Supplement: Supplementary file 1 — Additional file 1. Retrieved sequences used in this study. [file 12917_2022_3430_MOESM1_ESM.docx]

**Original un-cropped images and figures**

*
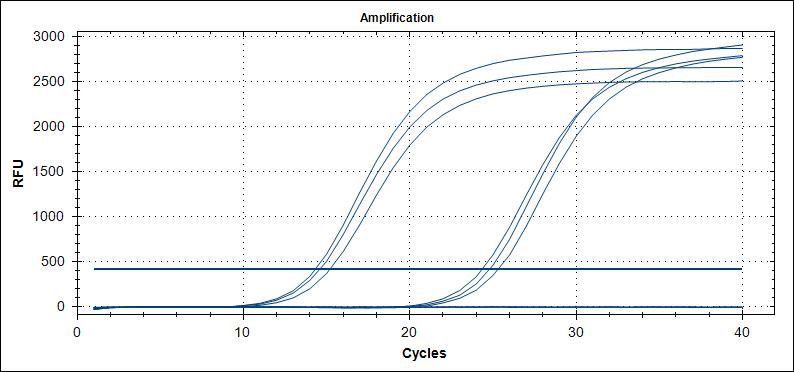
*

Figure 1: One-Step RT-PCR analysis. Amplification curves illustrating some of the select positive samples from different animals in both the Gatsibo and Nyagatare regions.

*
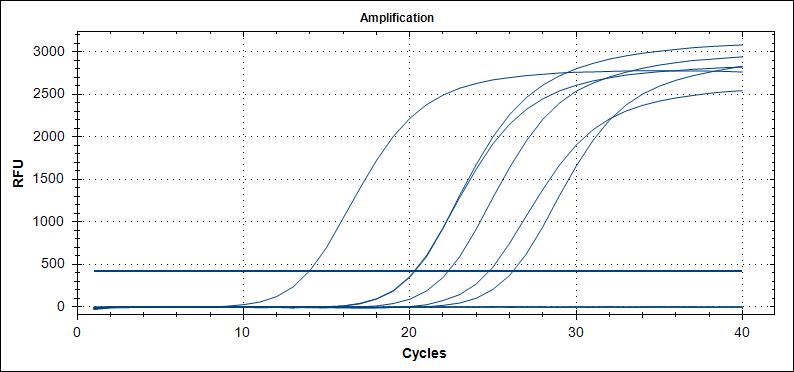
*

Figure 2: Amplification curves illustrating the detection of FMD viral RNA in the reaction, using OIE recommended TaqMan probes. The early triplicates were from sample 8 (Gatsibo) while the later threshold triplicates were from sample 26 (Nyagatare). The non-tem


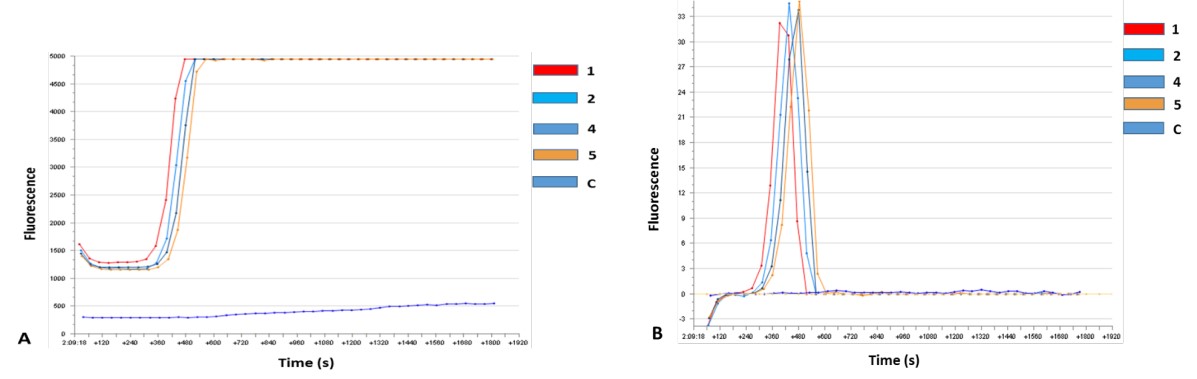


Figure 3: RT-LAMP results of field samples. Fig. 3.a describes the time trial of fluorescence detection of field samples and fig. 3.b shows the second derivative graphs of the fluorescence of the same samples collected in Eastern Rwanda during an outbreak.


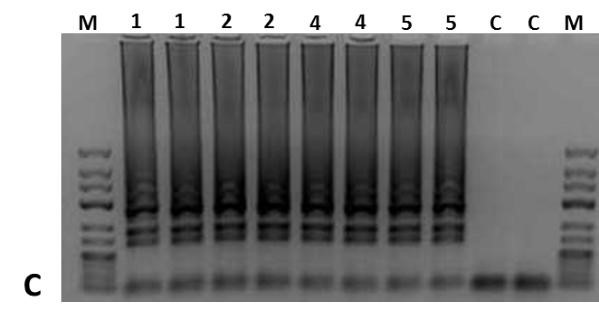


Figure 4: Agarose gel detection of RT-LAMP amplicons of selected PCR FMD positive duplicate samples from Nyagatare and Gatsibo.
