# Supplementary material for: Investigation of foot and mouth disease virus and other animal pathogens in cattle, buffaloes and goats at the interface with Akagera National Park 2017 – 2020
Source: BMC Vet Res. 2022 Sep 16;18:349. doi: 10.1186/s12917-022-03430-1 (PMC9479285; doi:10.1186/s12917-022-03430-1)
Supplement: Supplementary file 2 — Additional file 2. FMD serological NSP results from cattle and goats in Eastern Rwanda. [file 12917_2022_3430_MOESM2_ESM.docx]

**Select FMDV SAT prototype sequences and the branch length for Bayesian phylogenetic analysis.**

| **S/N** | **Isolate** | **Branch length** |
| --- | --- | --- |
| 1 | ZIM/14/2002 | 0.1275095 |
| 2 | MOZ/P13/2010 B16 | 0.08318924 |
| 3 | KEN/1/84 | 0.06441059 |
| 4 | ETH/1/90 | 0.04864008 |
| 5 | BOT/29/98 | 0.1183678 |
| 6 | ZIM/7/83 | 0.1130243 |
| 7 | NIG/2/75 | 0.06405975 |
| 8 | GHA/2/90 | 0.06299157 |
| 9 | UGA/51/75 | 0.09702656 |
| 10 | GAM/8/79 | 0.1563942 |
| 11 | ETH/2/91 | 0.1138175 |
| 12 | ETH/2/2007 | 0.06855722 |
| 13 | SUD/6/77 | 0.1115688 |
| 14 | RWA/1/00 | 0.1241663 |
| 15 | KEN/2/84 | 0.1182113 |
| 16 | UGA/19/98 | 0.09838044 |
| 17 | ZAI/1/82 | 0.1126227 |
| 18 | SAU/6/00 | 0.03160451 |
| 19 | CAR/8/2005 | 0.05841095 |
| 20 | ANG/4/74 | 0.118393 |
| 21 | UGA-BUFF/27/70 | 0.253833167 |
| 22 | ZIM/P25/91-UR-7 | 0.2211004 |
| 23 | RWAN/17 | 0.007234545 |
| 24 | RWAG/17 | 0.008035767 |
| 25 | NIG11/75 | 0.07706354 |
| 26 | ETH/3/2007 | 0.1229861 |
| 27 | TCH/1/72 | 0.1403647 |
| 28 | SUD/3/76 | 0.07947513 |
| 29 | UGA/13/74 | 0.1593325 |
| 30 | UGA/1/97 | 0.2168268 |
| 31 | ANG/9/74 | 0.1838095 |
| 32 | UGA-BUFF/21/70 | 0.07384378 |
| 33 | MOZ/1/75 | 0.1676978 |
| 34 | ZIM/23/2003 | 0.07887847 |
| 35 | T155/71 | 0.08837712 |
